# First insights into diversity and potential metabolic pathways of bacterial and fungal communities in the rhizosphere of *Argemonemexicana* L. (Papaveraceae) from the water-level-fluctuation zone of Wudongde Reservoir of the upper Yangtze river, China

**DOI:** 10.3897/BDJ.11.e101950

**Published:** 2023-08-08

**Authors:** Lanfang Zhou, Shengjun Wu, Maohua Ma

**Affiliations:** 1 School of River and Ocean Engineering, Chongqing Jiaotong University, Chongqing, China School of River and Ocean Engineering, Chongqing Jiaotong University Chongqing China; 2 Key Laboratory of Reservoir Aquatic Environment, Chongqing Institute of Green and Intelligent Technology, Chinese Academy of Sciences, Chongqing, China Key Laboratory of Reservoir Aquatic Environment, Chongqing Institute of Green and Intelligent Technology, Chinese Academy of Sciences Chongqing China; 3 Chongqing School, University of Chinese Academy of Sciences, Chongqing, China Chongqing School, University of Chinese Academy of Sciences Chongqing China

**Keywords:** dominant plant, predicted metabolic pathway, water-level fluctuation zone, rhizosphere microbial diversity, Wudongde reservoir

## Abstract

The water-level fluctuation zone (WLFZ) of Wudongde reservoir of the upper Yangtze river is a completely new aquatic-terrestrial transitional zone, and its plant degenerate issue is attracting global concerns. Uncovering the unknown rhizosphere microbiome of dominant plants of this zone is helpful in understanding the plant-microbe interactions and their growth under the largely varying environment. Here, a first exploration of the rhizosphere bacterial and fungal communities of wilted (JB) and unwilted (JA) *Argemonemexicana* L. individuals from the WLFZ of Wudongde reservoir was carried out using high-throughput sequencing and MetaCyc metabolic pathway analyses. The results showed that rhizosphere of wilted *A.mexicana* L individuals exhibited a higher microbial richness and diversity than the unwilted ones, irrespective of the bacterial and fungal communities. It was noted that 837 common bacterial amplicon sequence variants (ASV) and 92 common fungal ASV were presented in both JA and JB with 3108 bacteria and 212 fungi unique to JA, and 3569 bacteria and 693 fungi unique to JB. Linear discriminant analysis effect Size (LEfSe) analyses indicated that the taxa that had the most contribution to observed differences between both JA and JB was Proteobacteria, Actinobacteria and Ascomycota for JA, and Bacteroidetes, Firmicutes, Verrucomicrobia, Basidiomycota and Ascomycota for JB. Organic compound conversion pathway (degradation/reduction/oxidation) was consistently highly represented in the rhizosphere microbiomes of both JA and JB. Overall, this study provides insights into the rhizosphere microbiome composition, diversity and metabolic pathways of both wilted and unwilted *A.mexicana* L. individuals in the WLFZ of Wudongde reservoir, and the results give valuable clues for manipulating microbes to support plant growth in such a recently-formed WLFZ under a dry-hot valley environment.

## Introduction

Wudongde reservoir is located at the junction of Huidong County, Sichuan Province and Luquan County, Yunnan Province, and formed after the launch of Wudongde Hydropower Station as one of the world’s largest hydropower stations (Zhao et al. 2018, Dou et al. 2022). After finishing formal impounding in 2021, the water level generally varies between 952 m and 975 m, which leads to the formation of the water-level-fluctuation zone (WLFZ). The newly formed WLFZ is characterized by harsh environmental conditions (Niu et al. 2015), including typical dry-hot valley climate with a large evaporation capacity, long-term waterlogging stress and soil erosion. Under such environmental conditions, plants find it very difficult to survive in the WLFZ. They have to find effective strategies to adapt to the harsh environment. Some plants’ rhizosphere microbes have been extensively reported to have key roles in supporting their survival and defense (Korenblum et al. 2022, Ling et al. 2022, Liu et al. 2020). However, until now, no studies have reported the composition, diversity and function of rhizosphere microbial communities from the dominant plants in the WLFZ of Wudongde reservoir, the upper Yangtze river.

*Argemonemexicana* L. is a dominant plant in a part of the area of Wudongde WLFZ. *Argemonemexicana* L. is an erect prickly annual herb belonging to the family Papaveraceae (order Ranunculales) (Brahmachari et al. 2013, Manalil and Chauhan 2021). It distributes widely in the world, and often is the only cover of roadsides and fields. In particular, it can be used to treat a number of diseases such as tumors, microbial infection and inflammations. Its rhizosphere microbes in the WLFZ have not been explored up to now. Rhizosphere is an important zone of plant-microbe interactions that contributes to plant growth and adaption to various environmental stresses (Kong et al. 2022, Yang et al. 2020). Previous studies have demonstrated that rhizosphere seems to be able to attract a wide range of bacterial and fungal communities, and is associated with plant species (Ye et al. 2021). These microbes can help provide available nutrients for plant uptake or inhibit the plant pathogens (Gkarmiri et al. 2017, Hassan et al. 2019). Therefore, they are also called the plants’ second genome (Yuan et al. 2018). However, not all rhizosphere microbes are beneficial to the associated plants, *e.g.*, some deleterious microorganisms can cause plant diseases, inhibit plant growth and compete with nutrients. It appears that the microbial assembly in the plants’ rhizosphere is closely related to the plant traits and growth state (Ye et al. 2021). Thus, making a comparison of microbial community structures between both wilted and unwilted individuals from the same kind of plant at the same site may be helpful in understanding the plant-microbe interactions in the rhizosphere of dominant plants in the WLFZ of Wudongde reservoir.

In this study, high-throughput sequencing technology by an Illumina Novaseq platform together with PICRUSt software was employed to reveal the differences in rhizosphere bacterial and fungal communities between both wilted and unwilted *A.mexicana* L individuals from the WLFZ of Wudongde reservoir, the upper Yangtze river. High-throughput sequencing is a widely used technology for identifying the features of rhizosphere microbes in various plants in previous studies (Chaudhary et al. 2021, Liu et al. 2020, Wang et al. 2020, Xu et al. 2020, Yang et al. 2020, Liu et al. 2023), while PICRUSt software (Douglas et al. 2020) has been extensively applied to explore the functional profiles of rhizosphere microbes in Miscanthus (Chen et al. 2020), Gannan Navel Orange (Zhou et al. 2021), *Glycyrrhizauralensis* Fisch (Dong et al. 2021), *Cucurbitapepo* L. (Wang et al. 2022) and *Suaedasalsa* (Yu et al. 2022). The present study aims at providing a theoretical basis for understanding the interaction between dominant plants from the WLFZ and their rhizosphere microbes under a dry-hot valley climate and largely varying environment.

## Materials and methods

### Study area

The Jinsha River is a critical component of the Yangtze River (Su et al. 2022). Wudongde dam was established on the Jinsha River for the Wudongde hydropower station, crossing the left bank of Huidong county (Sichuan Province) and the right bank of Luquan county (Yunan Province) (Zhao et al. 2018). The study area is located at the WLFZ of Wudongde reservoir, Jinsha River Basin, Xinmin Village, Jiyi Town, Wuding County, Chuxiong Yi Autonomous Prefecture, Yunnan Province (26.1667° N, 102.2167° E). Monthly average maximum temperature, minimum temperature and precipitation in this area during 1981-2010 were 16.2-26.3 °C, 0.3-17.5 °C and 12.1-198.2 mm, respectively, according to Chinese Central Meteorological Station. This area belongs to the low-latitude plateau subtropical monsoon climate with sufficient sunlight and strong evaporation (Niu et al. 2015). With the Wudongde hydropower fully functioning in 2021, the water level changes between 952 m and 975 m in the Wudongde reservoir. Due to long submergence time (about 270-330 days per year), the vegetation coverage is very low in this area, where it was found that *A.mexicana* L. is a dominant plant in the present study area.

### Sampling

**Figure 1.**
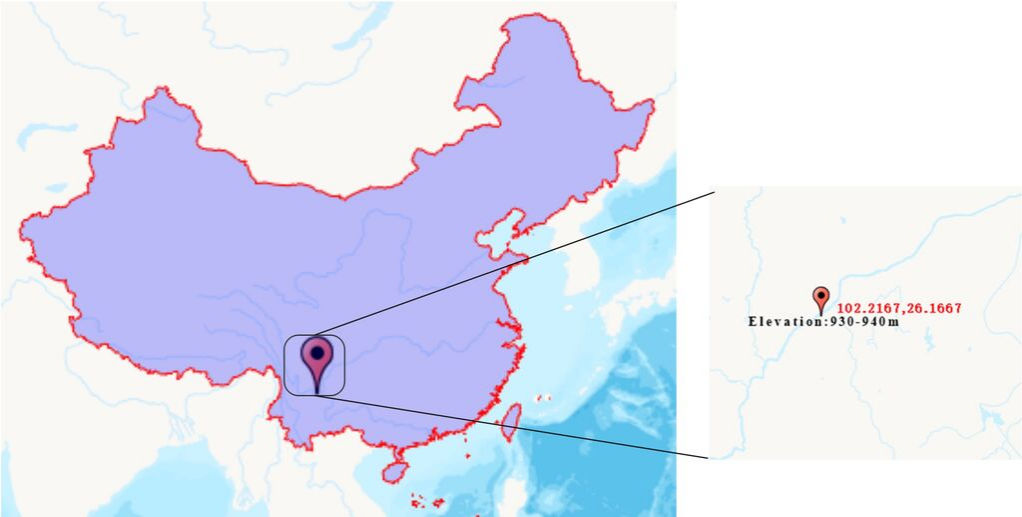
Sampling site.

The date for collecting the samples of rhizophere from A.mexicana was April 17, 2022. The rhizosphere soil samples were obtained from both wilted and unwilted *A.mexicana* L. individuals (each with more than 4 individuals) at different elevations and sites from the WLFZ by shaking the roots to make the adhering soil go into the sterile centrifuge tubes. Because the available amount of rhizosphere soil for a *A.mexicana* L. individual is very little each sample often requires sampling the rhizosphere soils from multiple *A.mexicana* L. individuals. All samples were obtained between elevations of 930-940 m (Fig. 1). Wilted and unwilted states can be observed for many plants. These states were assessed by plants’ growth performance, where the plant growth that is normal is called an unwilted state and the plant growth with the stem and leaf atrophying and bending toward the ground is called a wilted state. These soil samples from multiple wilted and unwilted *A.mexicana* L. individuals were mixed, respectively, with each having three replicates, and then stored at -80°C refrigerator before formal sequencing. For simplicity, the rhizosphere microbial samples from wilted and unwilted *A.mexicana* L. individuals were defined as JB and JA, respectively. Finally, genomic DNA of these samples was extracted by CTAB method.

### Data analyses

The amplification of 16S V3-V4 regions and ITS regions of each sample was carried out using primers of CCTAYGGGRBGCASCAG and GGACTACNNGGGTATCTAAT, and primers of CTTGGTCATTTAGAGGAAGTAA and GCTGCGTTCTTCATCGATGC, respectively. TruSeq® DNA PCR-Free Sample Preparation Kit (Illumina, USA) was adopted for the generation of sequence libraries. The Qubit@ 2.0 Fluorometer (Thermo Scientific) was used to analyze the library quality. The sequencing of rhizosphere microbes was performed using the Illumina NovaSeq platform with 250 bp paired-end reads produced. QIIME2 dada2 plugin was employed for the filtration, trim, denoising, and merging of sequences from each sample to get the feature table of amplicon sequence variant (ASV). The raw data of all samples were deposited in BioProject database (ID: PRJNA985046). Taxonomic information was obtained using the Greengenes (16S) and UNITE (ITS) databases based on sklearn algorithm. A wide number of bacteria and fungi were detected in the rhizosphere soil from *A.mexicana* L. under wilted and unwilted states, where unclassified microbes are removed for further exploration from the analyzed data. The bacterial and fungal compositions at phylum, class, order and genus levels were investigated. Chao1, Goods_coverage, Observed_features, Faith_pd, Simpson and Shannon indexes of bacterial and fungal communities were calculated to explore the alpha diversity. Finally, the functional profiles of rhizosphere microbiome were predicted using PICRUSt2 software with the MetaCyc metabolic pathways.

## Results and discussion

The interactions between dominant plants and their rhizosphere microbes are important for the growth of plants (Korenblum et al. 2022). However, the structure of bacterial and fungal communities in the rhizosphere soils of dominant plants in a recently formed WLFZ of Wudongde reservoir due to the construction of the world’s top ten hydropower (Wudongde hydropower) is still unclear. To answer this issue, *A.mexicana* L. is adopted as the representative of dominant plants of Wudongde reservoir WLFZ in this study. Accordingly, high-throughput sequencing based on the 16s rRNA or ITS sequences has been confirmed to be reliable, fast and low cost for determining the soil microbial features compared to traditional culture method, DGGE, T-RFLP and FISH (Li et al. 2021, Song et al. 2017, Yang et al. 2020). Thus, in this study, high-throughput sequencing was used to compare the rhizosphere microbial characterizations between both wilted and unwilted individuals (JB and JA).

### Richness and diversity of rhizosphere microbes

**Figure 2.**
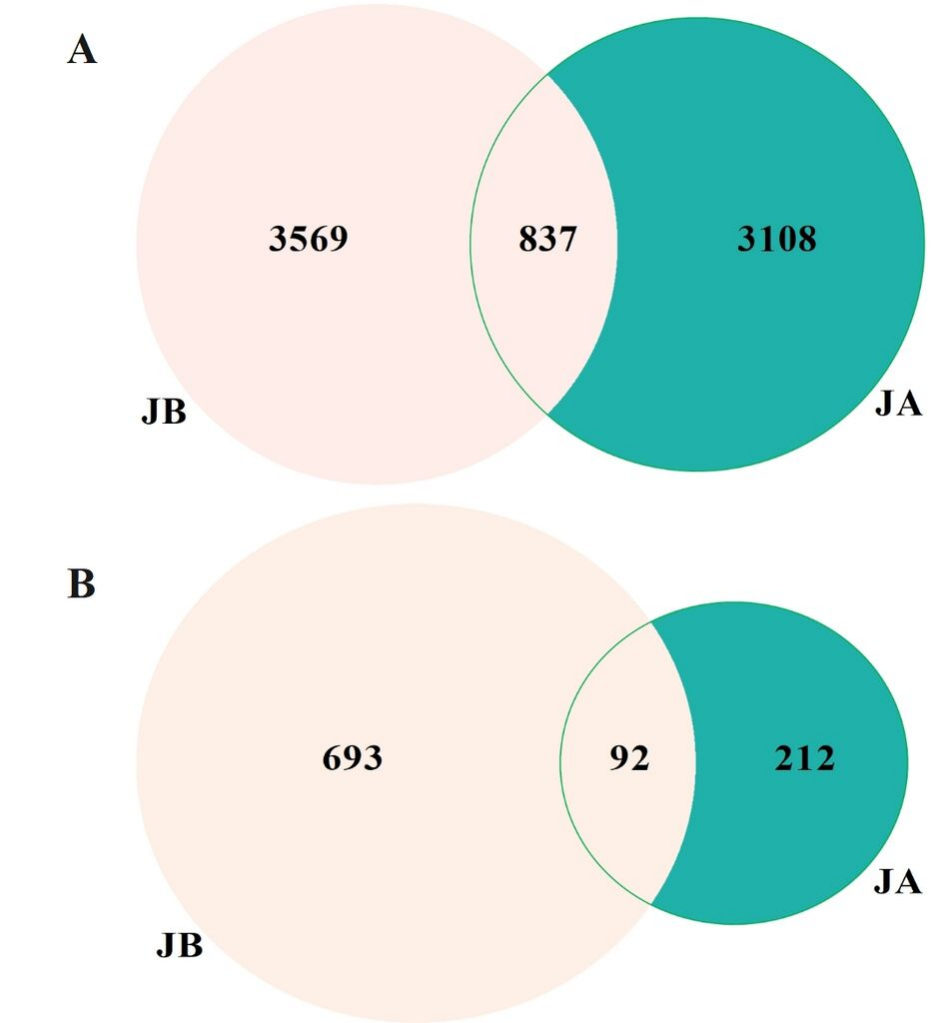
Venn diagrams of bacterial (A) and fungal (B) communities in the rhizosphere soils of both unwilted (JA) and wilted (JB) individuals of *A.mexicana* L. in the WLFZ.

In this study, a total of 500375 reads (range: 75440-87185, mean: 83396) for rhizosphere bacteria and 482739 reads (range: 60818-87130, mean: 80457) for rhizosphere fungi were obtained after sequencing. The Venn diagrams of bacterial communities in JA and JB are shown in Fig. 2. It was noted that 837 common bacterial amplicon sequence variants (ASV) were presented in both JA and JB with 3108 unique to JA and 3569 unique to JB. The Venn diagram of fungal communities is shown in Fig. 2B with 212 unique to JA, 693 unique to JB, and 92 common between them. The microbial list is available in Suppl. material 1.

**Figure 3.**
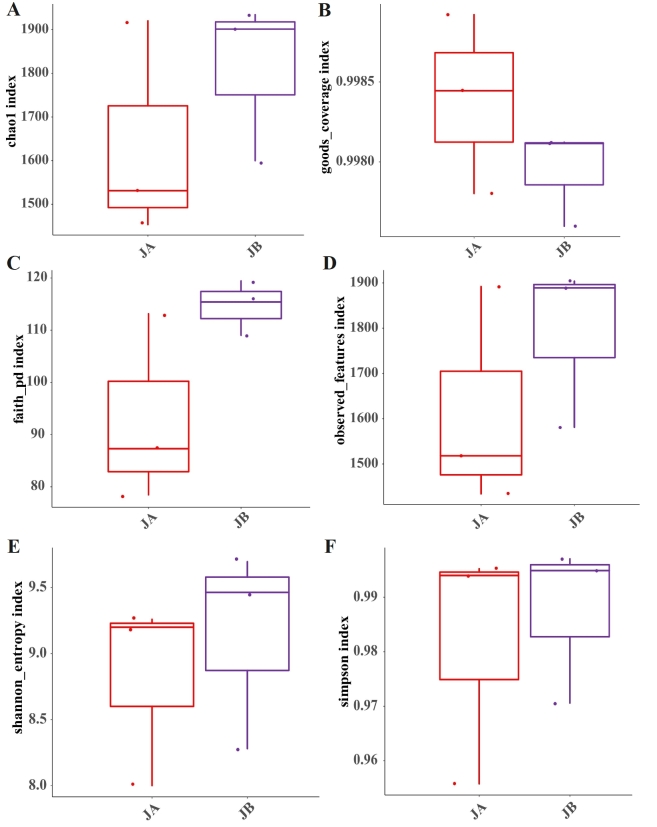
Bacterial alpha diversity indexes of the rhizosphere soils of both unwilted (JA) and wilted (JB) individuals of *A.mexicana* L. in the WLFZ. A, Chao1 index; B, goods_coverage index; C, Faith_pd index; D, Observed_features index; E, Shannon index; F, Simpson index.

Bacteria play an important role in soils, including organic matter decomposition, elemental cycles, plant symbiosis, etc. (Song et al. 2017). Clarifying the diversity in soil bacterial communities is becoming increasingly important in soil microecology. In previous studies, alpha diversity indexes of microbes were often used to reveal the richness and diversity of soil microbes (Khan et al. 2020, Man et al. 2020, Tong et al. 2021). In this study, the bacterial richness and diversity of JA and JB from *A.mexicana* L. are thus analyzed and shown in Fig. 3. The Chao1 and Observed_features indexes in JA are 1634.93±204.11 and 1614.67±199.08, respectively, being lower than JB where they are 1812.35±150.49 and 1791.33±148.85, respectively. This indicated that the wilted individuals have higher richness than the unwilted ones (Zhang et al. 2017). The same is true for Shannon and Simpson indexes, they are 8.82±0.58 and 0.98±0.02 in JA, being lower than JB with a Shannon index of 9.15±0.62 and a Simpson index of 0.99±0.01. Lower Shannon and Simpson indexes found in JA than in JB suggested less bacterial diversity in JA (Chen et al. 2020, Li et al. 2021). In terms of mean value of Faith_pd, there is JA<JB. Thus, it can be speculated that the phylogenetic diversity of bacterial species in JB is more complex than in JA (Faith 1992).

**Figure 4.**
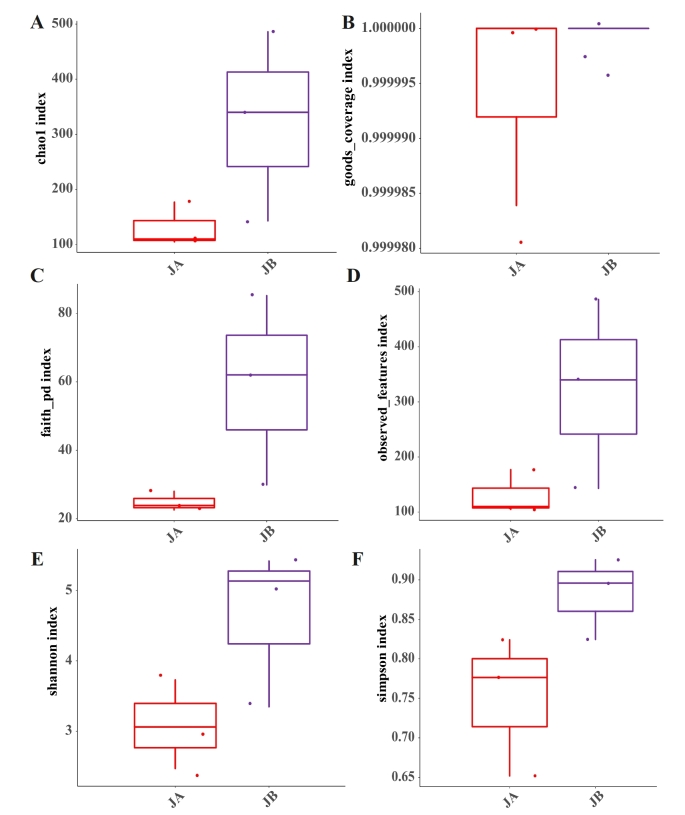
Fungal alpha diversity indexes of the rhizosphere soils of both unwilted (JA) and wilted (JB) individuals of *A.mexicana* L. in the WLFZ. **A** Chao1 index; **B** goods_coverage index; **C** Faith_pd index; **D** Observed_features index; **E** Shannon index; **F** Simpson index.

Soil fungi can act as biological controllers, decomposers and ecosystem regulators (Frąc et al. 2018). Fungal diversity is affected by plant traits, and in return regulate plant growth by symbiosis, pathogenicity and transformation. In this study, the rhizosphere fungal diversity of *A.mexicana* L. was thus investigated. Fig. 4 shows the fungal richness and diversity of JA and JB from *A.mexicana* L. It was found that the Chao1 and Observed_features indexes were consistent in JA (130.67±32.83), which were lower than those in JB (323±140.54). The Shannon and Simpson indexes in JB are 1.54 and 0.13 more than those in JA, respectively. Noteworthy is thatthe Faith_pd in JB is 24.21 more than that in JA. This suggested that the phylogenetic diversity of fungal community for evolution is higher in JB than in JA.

In summary, the coverage of both fungal and bacterial community in JA and JB is near 100%, characterized by goods_coverage index as shown in Figs 3, 4. The rhizosphere of wilted *A.mexicana* L. individuals has a higher microbial richness and diversity than the unwilted ones, irrespective of the bacterial and fungal communities. These findings suggest that the diversity of both fungal and bacterial community from the rhizosphere of *A.mexicana* L. has been affected by its wilted state. This may be because the rhizosphere microenvironment of *A.mexicana* L. has transformed under the wilted state as compared to the unwilted state.

### Relative abundance of rhizosphere bacteria

**Figure 5.**
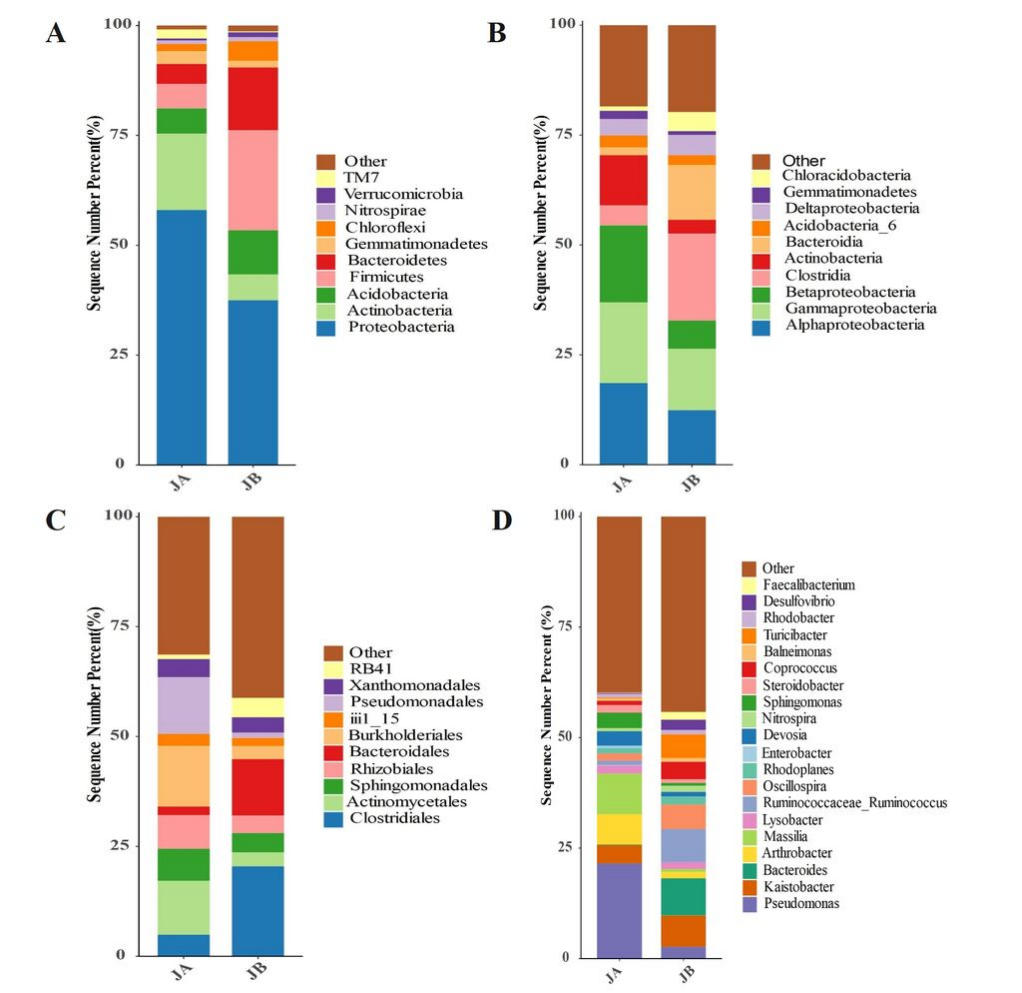
Rhizosphere bacterial community composition of both unwilted (JA) and wilted (JB) individuals of *A.mexicana* L. at the phylum (A), class (B), order (C) and genus (D) levels in the WLFZ, characterized by sequence number (relative abundance). Different colors indicate the top 10 phyla, classes and orders, and the top 20 genera; the rest of the bacteria are shown as “other”.

The bacterial phylum, class, order and genus at top relative abundances in rhizosphere of *A.mexicana* L. in the WLFZ are shown in Fig. 5. In the rhizosphere soils of *A.mexicana* L., Proteobacteria are the most abundant phylum in both JA (58%) and JB (37.48%). Similar trend was also observed in a previous study where Proteobacteria phylum was the most dominant in the rhizosphere soils of cucumber (Li et al. 2021). The second most abundant phylum is Actinobacteria (17.42%) for JA and Firmicutes (22.80%) for JB. Actinobacteria is reported to be more abundant in soils than in other media (Barka et al. 2016, Goodfellow and Williams 1983). For per gram of soil, there are 10^6^-10^9^ Actinobacteria cells. Some Actinobacteria are pathogen, but have a relatively minor role in plant pathogenicity compared to other bacteria. Instead, many Actinobacteria are able to provide beneficial effects to plant growth by competing with plant pathogen, production of antibiotics and nutrient supply. Like Actinobacteria, Firmicutes is also a frequently examined bacterial phylum in the rhizosphere soils (Lee et al. 2021, Zhang et al. 2019). The species from this phylum are often used as biofertilizers for plant growth promotion, biocontrol agents for the inhibition of plant pathogens, and bioremediation agents for pollutant removal (Hashmi et al. 2020). The order of other dominant phyla in JA is Acidobacteria (5.71%) > Firmicutes (5.55%) > Bacteroidetes (4.52%) > Gemmatimonadetes (2.98%) > TM7 (2.04%) > Chloroflexi (1.60%) > Nitrospirae (0.74%) > Verrucomicrobia (0.54%), while that is Bacteroidetes (14.28%) > Acidobacteria (9.99%) > Actinobacteria (5.91%) > Chloroflexi (4.48%) > Gemmatimonadetes (1.49%) > Verrucomicrobia (1.15%) > Nitrospirae (0.90%) > TM7 (0.19%) in JB.

The rhizosphere bacterial class was dominated by Alphaproteobacteria (18.59%), Gammaproteobacteria (18.36%), Betaproteobacteria (17.50%), Actinobacteria (11.46%), Clostridia (4.53%), Deltaproteobacteria (3.67%), Acidobacteria_6 (2.76%), Gemmatimonadetes (1.91%), Bacteroidia (1.76%) and Chloracidobacteria (0.98%). Alphaproteobacteria exhibited several kinds of morphologies (Stalked, stellate and spiral), involving in various metabolic strategies such as nitrogen fixation, ammonia oxidation and photosynthesis (Williams Kelly et al. 2007). Unuofin et al. (2019) found that some Gammaproteobacteria such as *Stenotrophomonasmaltophila* BIJ16, *Pseudomonasaeruginosa* DEJ16, and *Pseudomonasmendocina* AEN16 could produce laccase and degrade phenolic and non-phenolic contaminants. Betaproteobacteria presents the interactions with fungi by lichens and endosymbionts (Degli Esposti et al. 2018). Differing from Alphaproteobacteria with symbiosis genes located in plamids, chromosomes or chromids, Betaproteobacteria has its symbiosis genes in plasmids. However, the bacterial abundance pattern in JB is totally different from that in JA. The top ten bacterial Class in JB are Clostridia (19.74%), Gammaproteobacteria (13.94%), Alphaproteobacteria (12.43%), Bacteroidia (12.42%), Betaproteobacteria (6.44%), Deltaproteobacteria (4.62%), Chloracidobacteria (4.38%), Actinobacteria (3.17%), Acidobacteria_6 (2.25%) and Gemmatimonadetes (0.86%). It was reported that a number of compounds can be degraded by Clostridia, and it prefers strictly anaerobic or moderately aerotolerant environment (Dürre 2007).

It was found that the most dominant bacterial order is Clostridiales with a relative abundance of 20.45% in JB, but it only accounts for 4.93% in JA. In a previous study performed by Li et al. (2019), Clostridiales is also examined to be the most abundant order in pentachlorphenol-contaminated soils, and was critical to pentachlorophenol dichlorination. The addition of both citrate and lactate could modify the soil bacterial community with an increase of Clostridiales. Other dominant bacterial orders in JB are Bacteroidales (12.87%), Sphingomonadales (4.39%), RB41 (4.37%), Rhizobiales (3.98%), Xanthomonadales (3.55%), Actinomycetales (3.19%), Burkholderiales (2.89%), iii1_15 (1.94%) and Pseudomonadales (1.13%). In particular, the Bacteroidales dominating in JB only covers 1.95% in JA, and Bacteroidales has been considered a potential alternative to traditional fecal indicator bacteria for water quality monitoring in a previous study (Weidhaas et al. 2015). Burkholderiales (13.79%), Pseudomonadales (12.89%) and Actinomycetales (12.24%) occupied preponderance in the competition with many other bacteria in JA. It was also shown that Burkholderiales were enriched in maize-root soil samples, and harbored several beneficial effects on their linked plants, such as pathogen inhibition and nitrogen fixation (Aguirre-von-Wobeser et al. 2018). Both Rhizobiales (7.69%) and Sphingomonadales (7.28%) were also dominant in JA.

For the bacterial genus level, the abundance pattern between JA and JB is totally inconsistent. Compared to phylum, class, order, “other” groups in genus account for a very high percent in both JA and JB: >50% for JA and >60% for JB, if only top 10 bacteria were shown. Thus, we showed 20 top genera in this point. The most dominant genus *Pseudomonas* (21.57%) in JA only covers 2.66% in JB. *Pseudomonas* has been found to be able to increase the drought resistance of willows by enhancing nitrogen uptake (Kong et al. 2022).

**Figure 6.**
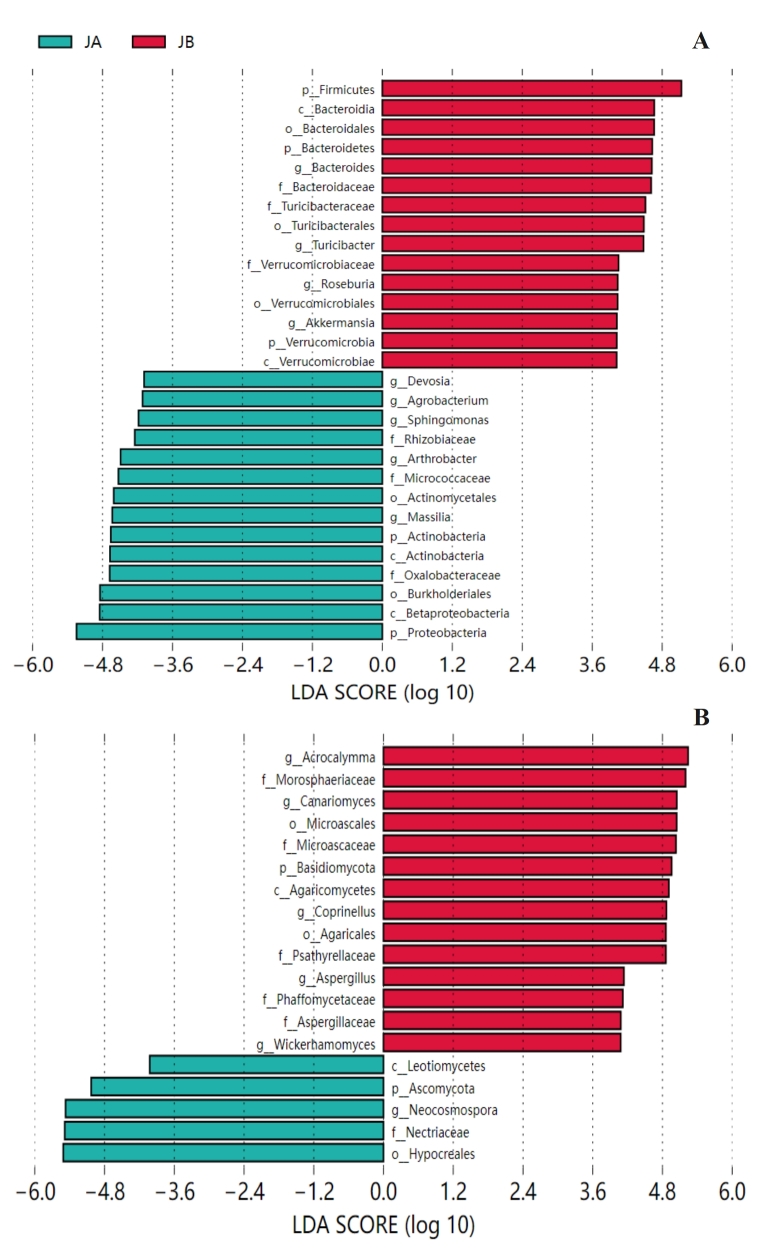
Linear discriminant analysis effect Size (LEfSe) analyses identify the taxa (phylum, class, order and genus) that have the most contribution to observed differences between JA and JB. **A** bacteria; **B** fungi. Relative abundance of bacteria or fungi is significant at P < 0.05 with a logarithmic LDA score threshold of 4.0.

The Linear discriminant analysis Effect Size (LEfSe) analyses were performed to explore the key bacterial taxa (genus level or higher) contribution mostly to the detected differences between JA and JB (Fig. 6A). It should be noted that LEfSe analysis has been believed to be efficient for identifying soil microbes that significantly responded to karst rocky desertification progression (Qi et al. 2018), chilli pepper-banana rotation (Hong et al. 2020) and soil-plant compartments in grapevine (Martínez-Diz et al. 2019). The major bacterial groups examined in JA were Proteobacteria, *Massilia*, *Devosia*, Actinobacteria, Micrococcaceae, Rhizobiaceae, *Sphingomonas*, Oxalobacteraceae, *Agrobacterium*, Actinobacteria, *Arthrobacter*, Burkholderiales, Betaproteobacteria, Actinomycetales. As compared to JA, Bacteroidales, Turicibacteraceae, Turicibacterales, Bacteroidetes, Verrucomicrobiales, Verrucomicrobiaceae, Bacteroidia, Firmicutes, *Turicibacter*, Verrucomicrobiae, Bacteroidaceae, *Bacteroides*, Akkermansia, Verrucomicrobia and Roseburia are highly represented in JB. Among these significantly different taxonomical groups in JA, 9 belong to Proteobacteria phylum, and 5 belong to Actinobacteria phylum. None of the other taxonomical groups were detected in this study. These findings suggested significant variations in bacterial community composition in the unwilted state of *A.mexicana* L. only occurred in both Proteobacteria and Actinobacteria phyla as compared to the wilted state of *A.mexicana* L. However, this is not the situation for JB, where 5 for Bacteroidetes, 5 for Firmicutes and 5 for Verrucomicrobia among these significantly different taxonomical groups in JB.

### Relative abundance of rhizosphere fungi

**Figure 7.**
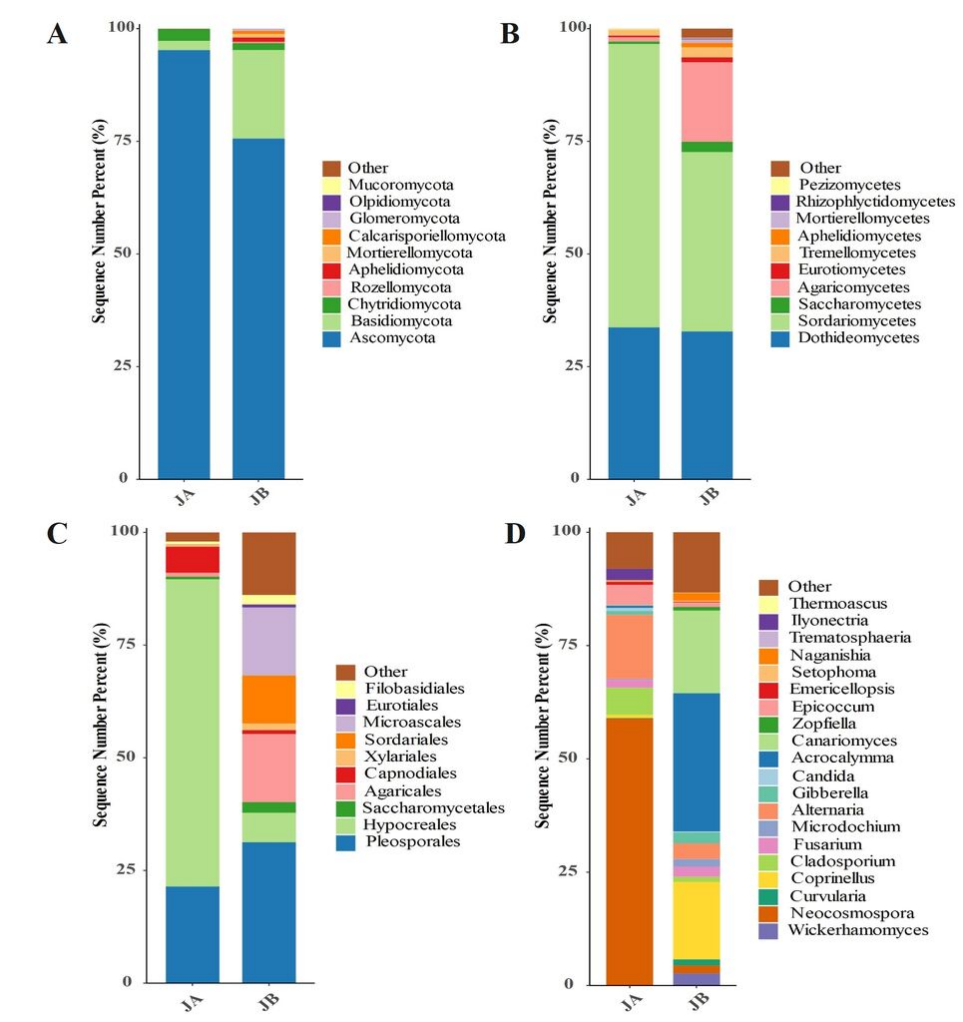
Rhizosphere fungal community composition of both unwilted (JA) and wilted (JB) individuals of *A.mexicana* L at the phylum (A), class (B), order (C) and genus (D) levels in the WLFZ, characterized by sequence number (relative abundance). Different colors indicate the top 10 phyla, classes and orders, and the top 20 genera; the rest of the bacteria are shown as “other”.

The fungal phylum, class, order and genus at top ten relative abundance are shown in Fig. 7. Ascomycota constituted the majority of fungal communities in the JA (95.24%). Chytridiomycota and Basidiomycota accounted for 2.65% and 2.04% of total fungal sequences, respectively. Other phyla indicated a relative abundance of < 0.04% in JA. Within JB samples, Ascomycota (75.61%) and Basidiomycota (19.63%) were the most abundant, followed by Chytridiomycota (1.54%) and Aphelidiomycota (1.01%), while the rest of the phyla were found to have a relative abundance less than 1%. Similar phenomena were also found in the rhizosphere soils of wheats (Gqozo et al. 2020), *Stipapurpurea* (Lu et al. 2016), potato (Hou et al. 2020), *Adeniumobesum*, and *Aloedhufarensis* (Khan et al. 2020), where Ascomycota and Basidiomycota were dominant. Ascomycota fungi have wide ecological niches, being correlated with carbon and nitrogen cycles, plant biomass decomposition and pathogenesis (Challacombe et al. 2019), while Basidiomycota fungi contain major pathogen lineages and mushroom-forming species (Coelho et al. 2017). For class level, the maximum variation occurred in Sordariomycetes between JA and JB, with 62.81% in JA changed to 39.77% in JB. It has been reported that Sordariomycetes is well known as one of the largest classes of Ascomycota (Maharachchikumbura et al. 2016), being extensively presented in marine, freshwater and terrestrial ecosystem. Several species such as *Trichodermaviride*, *T.harzianum* and *Beauveriabassiana* belonging to Sordariomycetes are important biocontrol agents (Kaewchai 2009, Maharachchikumbura et al. 2016). The second obvious variation appeared in Agaricomycetes between JA and JB, with an increase of 0.85% in JA to 17.61% in JB. Many Agaricomycetes are composed of wood-decaying fungi that play an important role in the carbon cycle. White rot and brown rot fungi of Agaricomycetes can decompose each part of plants’ cell walls and depolymerize cellulose, respectively (Kersten and Cullen 2014). Dothideomycetes is comparable between JA (33.73%) and JB (32.83%).

The diversity of fungal orders was lower in JA than in JB. Both Hypocreales (68.04%) and Pleosporales (21.49%) constituted almost 90% of all fungal sequences in JA. The order Hypocreales was reported to be the best-selected biocontrol fungi source for suppressing the deleterious plant pests, and many of them in the rhizosphere environment are able to outcompete plant pathogens and produce the promoters for plant growth (Contreras-Cornejo et al. 2016, Kepler et al. 2017). In addition, Capnodiales (5.87%) is a unique fungal order with a relative abundance > 1%. Different from JA, there are four fungal orders that have a relative abundance > 10%: Pleosporales (31.27%), Agaricales (15.17%), Microascales (15.07%) and Sordariales (10.65%). It should be noted that many other fungal orders were relatively abundant in JB, such as Hypocreales (6.53%), Saccharomycetales (2.33%) and Filobasidiales (2.09%).

The same is true for fungal genera, where the high abundance is only concentrated on several genera in JA, including *Neocosmospora* (58.90%), *Alternaria* (14.22%), *Cladosporium* (6.02%) and *Epicoccum* (4.43%). *Neocosmospora* and *Alternaria* covers a wide range of species that belong to endophytes, pathogens and saprobes (Lou et al. 2013, Sandoval-Denis et al. 2019). They are ubiquitous in soils, water and air. It has been demonstrated that more than 268 metabolites could be produced from *Alternaria* fungi, of which some compounds have the properties of anti-microbes (Lou et al. 2013). *Cladosporium* spreads in a variety of terrestrial and marine ecosystems, and often develops symbiotic relationships with many plants (Bensch et al. 2015, Salvatore et al. 2021). *Epicoccum* can be found in soils, air and decaying vegetation, and secretes various secondary metabolites (antioxidant, antimicrobial, and anticancer compounds) for phytopathogens and biotechnological applications (Braga et al. 2018). Other genera were in a very low abundance in JA. The most dominant genera were *Acrocalymma* (30.47%), *Canariomyces* (18.25%) and *Coprinellus* (17.11%) in JB, while other many genera were in a relatively even distribution, e.g., *Wickerhamomyces*, *Neocosmospora*, *Fusarium*, *Microdochium*, *Gibberella* and *Naganishia*, with a relative abundance of 1.5%-3%.

To explore the fungal taxa that has the greatest contribution to the observed differences between JA and JB, the LEfSe analyses were carried out (Fig. 6B). The major taxa in fungal group detected in JA samples were Leotiomycetes, Hypocreales, Ascomycota, Neocosmospora and Nectriaceae. It is very interesting that the Ascomycota fungal composition at all analyzed four taxonomical levels (phylum, class, order, family and genus) in JA were different from JB. Whereas in JB, As shown in Fig. 6B, represented fungi were 1 phylum (Basidiomycota), 1 class (Agaricomycetes), 2 orders (Microascales and Agaricales), 5 families (Aspergillaceae, Psathyrellaceae, Morosphaeriaceae, Phaffomycetaceae and Microascaceae) and 5 genera (*Coprinellus*, *Canariomyces*, *Acrocalymma*, *Aspergillus* and *Wickerhamomyces*). These taxa belong to Basidiomycota or Ascomycota phylum.

### Predicted metabolic pathways of rhizosphere microbes

The functional profiles of bacterial and fungal communities were analyzed by predicting metabolic pathways using PICRUSt2 software (Douglas et al. 2020), i.e., the metabolic pathway abundances were explored by structured mappings of EC gene families to MetaCyc pathways. The top 20 MetaCyc pathways in JA or JB are shown in Tables 1, 2. MetaCyc is a frequently-adopted database of metabolic pathways from all domains of life in the functional analyses of rhizosphere soils (Caravaca et al. 2022, Jiménez et al. 2020), covering 2749 pathways in the recent version (Caspi et al. 2020).

A total of 18 metabolic pathways were observed to significantly differ between the bacterial communities of both JA and JB (ANOVA, p<0.01), where the degradation/reduction pathways were highly represented with 13 among them directly related to these processes involved in the nitrate reduction and the degradation of anaerobic aromatic compounds, catechol, creatinine, toluene, adenosine nucleotides, salicylate, 1,5-anhydrofructose, pyrimidine ribonucleosides, L-arabinose, adenosine nucleotides, and L-valine. For the rhizosphere fungi, 14 metabolic pathways were found to present significant difference between JA and JB (p<0.01), of which 7 pathways were involved in the degradation/oxidation/metabolism/reduction, 4 pathways were involved in biosynthesis, 2 pathways were correlated with interconversion, and 1 pathway was phospholipid remodeling (phosphatidylethanolamine, yeast). Also, a large scale of rhizosphere bacteriome structure and function analysis based on 557 pairs of published sequencing data for the rhizosphere and bulk soils showed that organic compound conversion pathway was highly enriched in the rhizosphere (Ling et al. 2022). This can be expected, since plants’ rhizosphere is the pool of root litter that requires the participation of degradation-related microbes.

Notably, each of the top 20 dominant bacterial metabolic pathways in JA have very approximate relative abundance with JB (Table 1), where biosynthesis pathways (12/20) are highly dominant. The biosynthesis pathways were also reported to be enriched in the rhizosphere microbiome of Barley (Ye et al. 2022) and *Carpobrotusedulis* (Caravaca et al. 2022). Interestingly, almost each pathway of these pathways exhibited an even relative abundance distribution (0.6-1.5%) in the rhizosphere bacterial communities. However, this situation related to relative abundance for fungi is totally different from that in bacterial communities (Table 2). The dominant fungal metabolic pathways in JA were Aerobic respiration I (cytochrome c) (7.10%) and Aerobic respiration II (cytochrome c) (yeast) (7.10%), followed by adenosine ribonucleotides de novo biosynthesis (3.74%), D-myo-inositol (1,4,5)-trisphosphate biosynthesis pathway (3.34%), Pentose phosphate pathway (non-oxidative branch) I (3.29%) and glyoxylate cycle (3.14%), and Superpathway of adenosine nucleotides de novo biosynthesis I (2.44%), pyruvate fermentation to isobutanol (engineered) (2.42%), TCA cycle II (plants and fungi) (2.41%), tRNA charging (2.32%) and L-valine biosynthesis (2.27%), Superpathway of adenosine nucleotides de novo biosynthesis II (2.22%) and Superpathway of L-serine and glycine biosynthesis I (2.16%) are also abundant. The palmitate biosynthesis I (type I fatty acid synthase) pathway is the most abundant (6.02%) in JB, but it disappeared in JA. The same is true for stearate biosynthesis III (fungi), mitochondrial NADPH production (yeast), Sucrose degradation III (sucrose invertase), Fatty acid β-oxidation VII (yeast peroxisome) and they are enriched in JB with abundances of 2.85%, 2.44%, 2.06% and 2.00%, respectively, but they disappeared in JA.

## Conclusions

The present study reveals the diversity of bacteria and fungi in the rhizosphere soils of *A.mexicana* L. in the WLFZ of Wudongde revervior, the upper Yangtze river, and points out that the specific bacteria and fungi differ under growth states (unwilted VS. wilted) of *A.mexicana* L. using the LEfSe analyses. The rhizosphere of wilted *A.mexicana* L. individuals harbors a higher microbial richness and diversity than the unwilted ones based on the Chao1 index, Observed_features, Shannon index and Simpson index, irrespective of the bacterial and fungal communities. Dominant rhizosphere bacteria are Proteobacteria, Firmicutes and Actinobacteria. The rhizosphere fungal communities are nearly completely occupied by Ascomycota in the unwilted individuals (95.3%), while a large proportion of fungal communities are shared by Basidiomycota (19.63%) besides the Ascomycota (75.61%) in the wilted individuals. Organic compound conversion pathway is highly represented in both wilted and unwilted *A.mexicana* L. individuals. The information on the composition, diversity and functions in the rhizosphere microbiomes of the dominant plants is critical to understand and manipulate their ecosystem functions to support the future plant growth in such a typical ecological vulnerable zone (WLFZ) in large reservoirs.

## Supplementary Material

79EDD323-58FD-5F37-AF3B-D271D306107B 10.3897/BDJ.11.e101950.suppl1 Supplementary material 1 Microbial list Data type Text File: oo_889597.pdf https://binary.pensoft.net/file/889597 Lanfang Zhou, Shengjun Wu, Maohua Ma

## Figures and Tables

**Table 1.** The top 20 dominant metabolic pathways in rhizosphere bacterial communities from the *A.mexicana* L. in the WLFZ of Wudongde reservoir.

**Full name for metabolic pathway**	**Abbreviation for metabolic pathway**	**JA**	**JB**
Aerobic respiration I (cytochrome c)	PWY-3781	1.50%	1.19%
Pyruvate fermentation to isobutanol (engineered)	PWY-7111	1.01%	0.85%
L-isoleucine biosynthesis II	PWY-5101	0.89%	0.85%
L-isoleucine biosynthesis I (from threonine)	ILEUSYN-PWY	0.86%	0.81%
L-valine biosynthesis	VALSYN-PWY	0.86%	0.81%
Cis-vaccenate biosynthesis	PWY-5973	0.73%	0.75%
Superpathway of branched chain amino acid biosynthesis	BRANCHED-CHAIN-AA-SYN-PWY	0.74%	0.73%
Gondoate biosynthesis (anaerobic)	PWY-7663	0.70%	0.75%
CDP-diacylglycerol biosynthesis I	PWY-5667	0.72%	0.71%
CDP-diacylglycerol biosynthesis II	PWY0-1319	0.72%	0.71%
Pentose phosphate pathway (non-oxidative branch) I	NONOXIPENT-PWY	0.65%	0.76%
Fatty acid elongation -- saturated	FASYN-ELONG-PWY	0.65%	0.67%
L-isoleucine biosynthesis III	PWY-5103	0.67%	0.67%
Superpathway of phospholipid biosynthesis I (bacteria)	PHOSLIPSYN-PWY	0.66%	0.66%
TCA cycle	TCA	0.66%	0.62%
TCA cycle V (2-oxoglutarate synthase)	PWY-6969	0.62%	0.63%
Phosphatidylglycerol biosynthesis I	PWY4FS-7	0.62%	0.63%
Phosphatidylglycerol biosynthesis II	PWY4FS-8	0.62%	0.63%
Fatty acid salvage	PWY-7094	0.74%	0.48%
Superpathway of pyrimidine nucleobases salvage	PWY-7208	0.62%	0.66%

**Table 2.** The top 20 dominant metabolic pathways in rhizosphere fungal communities from the *A.mexicana* L. in the WLFZ of Wudongde reservoir

**Full name for metabolic pathway**	**Abbreviation for metabolic pathway**	**JA**	**JB**
Palmitate biosynthesis I (type I fatty acid synthase)	PWY-5994	0.00%	6.02%
D-myo-inositol (1,4,5)-trisphosphate biosynthesis	PWY-6351	3.34%	3.31%
Glyoxylate cycle	GLYOXYLATE-BYPASS	3.14%	3.04%
Stearate biosynthesis III (fungi)	PWY3O-355	0.00%	2.85%
Adenosine ribonucleotides de novo biosynthesis	PWY-7219	3.74%	2.29%
Pyruvate fermentation to isobutanol (engineered)	PWY-7111	2.42%	2.30%
Mitochondrial NADPH production (yeast)	PWY-7269	0.00%	2.44%
Trna charging	TRNA-CHARGING-PWY	2.32%	2.22%
TCA cycle II (plants and fungi)	PWY-5690	2.41%	2.21%
L-valine biosynthesis	VALSYN-PWY	2.27%	2.09%
Chitin deacetylation	PWY-7118	2.27%	2.11%
Superpathway of adenosine nucleotides de novo biosynthesis I	PWY-7229	2.44%	2.06%
Superpathway of L-serine and glycine biosynthesis I	SER-GLYSYN-PWY	2.16%	2.05%
Pentose phosphate pathway (non-oxidative branch) I	NONOXIPENT-PWY	3.29%	1.96%
Fatty acid β-oxidation VII (yeast peroxisome)	PWY-7288	0.00%	2.00%
GDP-mannose biosynthesis	PWY-5659	1.92%	1.94%
Superpathway of adenosine nucleotides de novo biosynthesis II	PWY-6126	2.22%	1.92%
Sucrose degradation III (sucrose invertase)	PWY-621	0.00%	2.06%
Aerobic respiration I (cytochrome c)	PWY-3781	7.10%	1.56%
Aerobic respiration II (cytochrome c) (yeast)	PWY-7279	7.10%	1.56%
