# Supplementary material for: First insights into diversity and potential metabolic pathways of bacterial and fungal communities in the rhizosphere of Argemonemexicana L. (Papaveraceae) from the water-level-fluctuation zone of Wudongde Reservoir of the upper Yangtze river, China
Source: Biodivers Data J. 2023 Aug 8;11:e101950. doi: 10.3897/BDJ.11.e101950 (PMC10848652; doi:10.3897/BDJ.11.e101950)
Supplement: Supplementary material 1 — Microbial list [file bdj-11-e101950-s001.pdf]

Acidobacteria

Acrocalymma

Actinobacteria

Actinomycetales

Agaricales

Alphaproteobacteria

Alternaria

Aphelidiomycota

Ascomycota

Bacteroidales

Bacteroidia

Basidiomycota

Betaproteobacteria

Burkholderiales

Canariomyces

Capnodiales

Chloracidobacteria

Chloroflexi

Chytridiomycota

Cladosporium

Clostridia

Clostridiales

Coprinellus

Deltaproteobacteria

Dothideomycetes

Epicoccum

Filobasidiales

Firmicutes

Fusarium

Gammaproteobacteria

Gemmatimonadetes

Gibberella

Hypocreales

Microascales

Microdochium

Naganishia

Neocosmospora

Nitrospirae

Pleosporales

Proteobacteria

Pseudomonadales

Pseudomonas

Rhizobiales

Saccharomycetales

Sordariales

Sordariomycetes

Sphingomonadales

Verrucomicrobia

Wickerhamomyces

Xanthomonadales
